# Systemic Inflammation in the Genesis of Frailty and Sarcopenia: An Overview of the Preventative and Therapeutic Role of Exercise and the Potential for Drug Treatments

**DOI:** 10.3390/geriatrics2010006

**Published:** 2017-01-17

**Authors:** Stephen C. Allen

**Affiliations:** 1 The Royal Bournemouth Hospital, Castle Lane East, Bournemouth, Dorset BH7 7DW, UK; Stephen.allen@rbch.nhs.uk; Tel.: +44-1202-704-539; Fax: +44-1202-704-542; 2 Centre for Postgraduate Medical Education and Research, Bournemouth University, Bournemouth, Dorset BH12 5BB, UK

**Keywords:** ageing, inflammation, cytokines, sarcopenia, frailty, exercise, anti-inflammatory drugs

## Abstract

The clinical, pathological and biological characteristics of frailty and sarcopenia are becoming better understood and defined, including the role of systemic inflammation. It is increasingly apparent that in older adults there is a tendency for the innate immune network to shift toward a pro-inflammatory setting, often due to the presence of chronic inflammatory diseases but also associated with age alone in some individuals. Furthermore, acute inflammation tends to resolve more slowly and less completely in many elderly people. Inflammation contributes to the pathogenesis of sarcopenia and other components of the frailty syndrome. Blood levels of inflammatory cytokines and acute phase proteins, are reduced by exercise, and there is a growing body of epidemiological, observational and intervention research that indicates that regular moderate exercise improves strength, function, morbidity and mortality in middle-aged and elderly adults. There is also an increasing awareness of the potential role of drugs to ameliorate inflammation in the context of frail old age, which might be particularly useful for people who are unable to take part in exercise programs, or as adjunctive treatment for those who can. Drugs that shift the innate immune biochemical network toward an anti-inflammatory setting, such as methyl-xanthines and 4-amino quinolones, could be of value. For example, theophylline has been shown to induce a 20 percent fall in pro-inflammatory tumor necrosis factor (TNF) and 180 percent rise in anti-inflammatory interleukin-10 production by peripheral blood monocytes, and a fall of 45 percent in interferon-gamma (IF-gamma) release. Such properties could be of therapeutic benefit, particularly to re-establish a less inflamed baseline after acute episodes such as sepsis and trauma.

## 1. Introduction

Geriatricians and other clinicians have become increasingly aware of the need to gain a better understanding of the frailty syndrome, a clinical state that can cause difficulties for elderly patients across the bio-psycho-social spectrum. The focus of this paper, set within the framework of old age, will be the key role of systemic inflammation in the pathogenesis of several aspects of frailty including sarcopenia and cachexia. The preventive and therapeutic utility of exercise in this context will be considered. There is now an extensive literature dealing with these, and closely related, topics so this review will be an overview of key insights and evidence over the last 25 years. With respect to the future, the possibility of preventing, ameliorating or reversing aspects of frailty by intervening with pharmacological agents will also be considered.

## 2. Defining Frailty

Frailty is now recognized as a deleterious phenotype encountered mainly in elderly people who are predisposed to a range of functional impairments when the reduced physiological reserves of old age are additionally compromised by superimposed disease. Definitions vary and are inevitably generalizations. A representative example of such definitions is as follows:

“A condition, seen particularly in older patients, characterized by low functional reserve, easy tiring, decrease of libido, mood disturbance, accelerated osteoporosis, decreased muscle strength, and high susceptibility to disease. People with the frailty syndrome may take a sudden turn for the worse and die. However, the frailty syndrome may sometimes be reversible”.

[1]

Any definition of frailty requires a number of qualifying statements, such as are embedded in the British Geriatrics Society published guidance [2] that begins:

“Frailty is a distinctive health state related to the ageing process in which multiple body systems gradually lose their in-built reserves. Around 10% of people aged over 65 years have frailty, rising to between a quarter and a half of those aged over 85 years. Older people living with frailty are at risk of adverse outcomes such as dramatic changes in their physical and mental wellbeing after an apparently minor event which challenges their health, such as an infection or new medication”.

Evidence-based work has been published to establish the characteristics of the frailty phenotype [3] and to develop an index for use in clinical practice [4].

Frailty is obviously not a categorical state but a spectrum running from pre-frail, when individuals are functioning well but are at high risk of becoming overtly frail, through to states of extreme frailty in which multiple physiological systems are failing, independence has been lost, and death is imminent. Some aspects of frailty are reversible, particularly in the pre-frail state and after acute illnesses. Some of the interventions discussed in this paper will focus on that potential for improvement.

## 3. Sarcopenia—A Frequent Component of Frailty

A large proportion of frail elderly people suffer from weakness of their skeletal muscles to an extent that impairs mobility, postural stability and independent living. Though this phenomenon is encountered with increasing frequency with age, it varies greatly in severity and is not present to a disabling degree in all aged people. There is a clear association with other markers of poor health, such as chronic inflammatory conditions, and higher rates of mortality [5], and there is now a wide consensus among clinicians and gerontologists that muscle wasting and weakness that leads to functional consequences should be regarded as pathological. It is that state of the skeletal muscles, in which there is a loss of mass and function, that is referred to as sarcopenia. Sarcopenia is now recognized as a specific condition and has recently been allocated an International Classification of Diseases-10 (ICD-10) code. It must be emphasized that sarcopenia is not synonymous with frailty but, like other causes of muscle weakness, is an important contributing component of frailty in a large proportion of patients.

There is no universally accepted definition of sarcopenia. This reflects the observation that the phenotype of sarcopenia appears to be a final common pathway arising from a wide range of pathological processes, often contributing in concert but in varying proportions in individual people, and superimposed on natural ageing changes.

In recent years, clinical definitions of sarcopenia have been developed, mainly to lend clarity to clinical practice, research, and studies on the epidemiology of the condition. Definitions generated by various groups have tended to converge and, as would be expected, focus on certain phenotypic characteristics. Though there is no single agreed definition, set of diagnostic criteria or treatment guideline, the best extant operational brief definition contends that sarcopenia is a syndrome characterized by progressive and generalized loss of skeletal muscle mass and strength with a risk of adverse outcomes such as physical disability, poor quality of life and death. This has emerged from consensus papers published by The European Working Group on Sarcopenia in Older People (EWGSOP), the European Society for Clinical Nutrition and Metabolism Special Interest Groups (ESPEN-SIG) and International Working Group on Sarcopenia (IWGS) [6,7,8]. Perhaps the consensus paper with the greatest utility is that of EWGSOP which defines sarcopenia as:

“The presence of a low skeletal muscle mass and either low muscle strength (e.g., handgrip) or low muscle performance (e.g., walking speed or muscle power); when all three conditions are present severe sarcopenia may be diagnosed”.

[6]

It was suggested that for subjects over the age of 65 years a walking speed below 0.8 m/s on a 4 m walking test should be considered abnormal. This could prompt measurement of muscle mass using dual energy X-ray absorptiometry (DEXA), which would be abnormally low if more than 2 standard deviations below the normal young mean (7.23 kg/m^2^ for men and 5.67 kg/m^2^ for women). Also, if the walking speed is more than 0.8 m/s the hand grip should be tested and if lower than 20 kg for women or 30 kg for men the muscle mass should be assessed by DEXA.

The EWGSOP definition [6] has the additional benefit of facilitating the sub-division or staging of sarcopenia, which could clearly be utilized for outcomes assessments when interventions are trialed. The stages defined are: Pre-sarcopenia—characterized as low muscle mass without an impact on muscle strength or physical performance. This would require accurate methods of muscle mass measurement and an established standard population reference. Sarcopenia—is defined as low muscle mass with low strength or low physical performance. Severe sarcopenia—the stage when all three are present (low muscle mass, low strength and low performance).

The Society of Sarcopenia, Cachexia and Wasting Disorders Trialist Workshop settled on a similar working definition [9], which also has international standing, qualifying it by stating that other causes of muscle wasting and/or weakness, such as neurological diseases or primary muscle disorders, should be excluded. Some researchers have understandably emphasized the need to separate sarcopenia from cachexia, the latter being usually defined as severe loss of body weight, fat and muscle loss and increased protein catabolism due to underlying disease, such as malignant tumors or chronic infection [9,10]. However, the loss of muscle mass and strength encountered in many cachectic patients would meet much of the clinical definition of sarcopenia described above, though the histological changes are not identical. Therefore, there is an important overlap that clinicians must take into account, particularly when treating patients with lower amplitude inflammatory states that predispose to sarcopenia yet share a number of pathogenic factors, and opportunities for intervention, with overt cachexia. As will be discussed later in this paper, persistent pro-inflammatory activity and dysregulation of the innate immune system is emerging as one of the most ubiquitous etiological factors in the genesis of sarcopenia and other aspects of the frailty syndrome, and have also been identified as key contributors to loss of appetite, under-nutrition and cachexia [10]. Furthermore, not all cachectic states are irreversible so a case can be made for the optimal management of cachexia to minimize the likelihood of sarcopenia after recovery.

There is relatively little disagreement on the histological changes that typify sarcopenia, and how they differ from disuse atrophy [11]. An age-related decline in the number of muscle fibers and their size is seen, particularly after the age of 65 years, and that change is exaggerated in sarcopenia. There is a loss of type 1 (slow-twitch) and type 2 (fast-twitch) fibers, though type 2 fibers tend to atrophy by 15–30 percent with aging. There is also increased fiber size variability and an accumulation of non-grouping, scattered and angulated fibers [12]. Fat tissue infiltration between muscle fibers increases with age. In one study the quadriceps femoris intramuscular fat was found to be 61 percent and 81 percent higher in elderly women and men respectively, compared to younger women and men. In the same study the fall in muscle volume was 27 and 26 percent respectively [13], Intramuscular fat is particularly excessive in subjects with obese sarcopenia, but no generally agreed definitions have been reached beyond a combination of an obese body mass index (>30 kg·m^−2^) combined with an adjusted grip strength below the third tertile [14]. There is no clear agreement on the etiological interpretation of the microscopic changes seen in sarcopenic muscle cells, and a full discussion of that topic is outside the scope of this paper. However, it is becoming increasingly likely that mitochondrial dysfunction is at least partly responsible for the accumulation of oxidative damage observed in ageing myocytes. Further, there is evidence that indicates that this might be a key factor contributing to the defective regulation of myocyte apoptotic signaling, as suggested by the increased myonuclear DNA fragmentation seen in aged muscle. Mitochondria are an important part of the process of apoptosis through their central role in cellular energy distribution [15].

The prevalence of sarcopenia is not really known in any population, and that is even more the case if pre-sarcopenia is considered. Quoted prevalence rates vary geographically, by the definition employed and by the sampling methodology. Some of the most reliable studies have indicated a prevalence of around 5%–13% in the 60–70-year-old age group and 11%–50% for those above the age of 80 years [16]. Even the most conservative prevalence data indicate that sarcopenia is common and widespread, and will inevitably increase as populations age.

## 4. Systemic Inflammation as a Cause of Frailty

Many studies have shown that systemic inflammation is closely associated with a number of aspects of frailty, including sarcopenia and cachexia [17,18,19]. The molecular mechanisms are clearly complex and it has been difficult to establish a reproducible and robust biochemical explanation to account for the full scope of observed effects. Perhaps the main reason for this is that components of the innate immune systems, particularly chemokine signaling, the catecholamine-cortisol system, complement cascades, interferons, immune-competent cells and other somatic cells communicate with, and influence, each other in a manner more akin to a network or web than a series of linear biochemical reactions. A change or intervention in one part of the web results in changes in other parts that are conditional upon multiple variables, thus resulting in an apparent lack of reproducibility of studies focused on a small range of molecules. It can consequently be argued that it might not be possible to establish generic mechanisms that explain the outcomes from interventions in individuals, so effective interventions might be more likely to be those that can impart broad corrective changes to the innate immune network, sometimes referred to as immune modulation, and thereby reduce the inflammatory burden that contributes to frailty.

## 5. Background Inflammation in Old Age

Next, we need to consider the state of the innate immune network in elderly people. It has been clearly established that baseline biochemical markers of inflammation, such as interleukin-1 (IL-1), tumor necrosis factor (TNF) and C-reactive protein (CRP) are raised 2–4-fold in the peripheral blood of older individuals, particularly those above the age of 80 years, compared to the levels found younger adults [20,21,22]. For example, a study of healthy people showed mean baseline blood TNF concentrations of 0.6 pg/mL in a sample of adults below the age of 30 years compared to 1.5 pg/mL in a sample above the age of 70 years [21]. A population study found the upper 95th centile for CRP to be age/50 and age/50 + 0.6 for white men and women respectively. The corresponding values for those of African descent were age/30 and age/30 + 1.0 [23]. This has been referred to as “inflammaging” by some authors [22]. In many cases this can be explained by the presence of a clinically apparent chronic inflammatory condition such as chronic obstructive pulmonary disease (COPD) or rheumatoid arthritis (RA) [24]. Similar levels of biochemical inflammation are often observed in elderly people with other disorders that are now known to shift the innate immune network toward a pro-inflammatory setting, such as central obesity, cardiovascular disease, or type 2 diabetes mellitus [25,26,27].

Of particular relevance to this paper is the augmented inflammatory state that is often present as a persisting or slowly resolving abnormality after an acute inflammatory event [28,29]. Raised pro-inflammatory biomarkers have also been associated with Alzheimer’s disease (AD), physical inactivity, chronic kidney disease and osteoarthritis [25,30,31,32]. In some clinically well elderly people raised biochemical inflammatory markers appear to occur with no demonstrable cause, and age itself appears to be associated with chronic inflammation [22,33,34,35]. A community study of healthy volunteers found the mean blood CRP to be 0.9 mcg/mL in young adults compared to 3.0 mcg/mL in people over the age of 65 [33]. IL-6 and IL-1 have been shown to be significantly correlated with age [35] However, not all studies have confirmed this observation in healthy older people [36,37]. Persisting low-amplitude inflammation is associated with increased all-cause mortality [38,39], reduced muscle strength [40,41], impaired instrumental function [39] and lower self-reported health status [42]. The observed levels of inflammatory markers, such as CRP, IL-1, IL-6 and TNF vary between studies, depending on the collection and assay methods used, but the adverse outcomes mentioned above were generally associated with 1.5 to 3-fold elevation above those found in healthy people of comparable age. There are few direct comparisons with young people in relation to functional outcomes or specific pathologies.

There is evidence that chronic disease progression is at least partly due to chronic inflammation and not merely indicated by it [43], so there is a complex interaction between cause and effect. The endothelial inflammation that occurs in atheromatous vascular disease is probably one of the most studied examples, and therefore is often regarded as a model for the interaction between ageing changes, inflammation and pathology [44]. It also appears that in many elderly people, the biochemical pro-inflammatory response to acute illnesses such as infection or injury does not resolve as quickly as it does in younger adults [28,29]. The rises in IL-1, IL-6 and TNF persists longer and the rise in the anti-inflammatory cytokine interleukin-10 (IL-10) is delayed and slower. The objective evidence for this effect has been most clearly established for pneumococcal and endotoxin antigens [45,46], indicating an approximate 2-fold difference in the time to return to baseline between young and older subjects, despite similar initial peak levels. This suggests an impairment of regulatory anti-inflammatory function that can be seen as a delay in re-setting the normative surveillance state of the innate immune network. That might be one of the reasons for the slower rates of clinical recovery and chronic low-level inflammation often seen in old age.

There are other purported mechanisms for chronic age-associated inflammation however, including a decline in anti-oxidant capacity with consequent increased oxidative stress [47], which appears to contribute to pro-inflammatory activity through stimulation of toll-like receptors on various immune cells [48], though from another perspective this can all be seen as part of the entirety of the immune dysfunction described above. A pragmatic argument can also be proposed that links raised baseline inflammation in old age to enhanced anti-neoplastic immune surveillance and sub-clinical auto-immune states. It is also not clear whether the tendency for cortisol and catecholamine levels to remain higher for longer after acute damage in old age [49] is entirely secondary to higher pro-inflammatory cytokine levels or partly due to non-cytokine mechanisms governed by the pituitary-adrenal axis. Nevertheless, the evidence for cytokines having a central role in age-associated inflammation is now beyond contention.

## 6. The Case for Inflammation as a Pathogenic Factor

The disturbance of inflammatory control and modulation described above appears to play an important part in the pathogenesis and perpetuation of a number of common and clinically significant conditions in old age. These include cardiovascular and cerebrovascular disease, metabolic syndrome, sarcopenia, some malignancies and neuronal dysfunction [24], all of which can contribute to the frailty phenotype. The effect on neuronal function is particularly subtle and poorly understood, but there is evidence that pro-inflammatory over-activity and inadequate or incomplete anti-inflammatory activity contributes to cognitive impairment, low mood, illness behavior and autonomic dysfunction, though the observed extent and magnitude of these effects vary greatly between studies [24].

Chronic low-level elevation of CRP, released from hepatocytes mainly in response to raised IL-1, IL-6 and TNF, is associated with enhanced risk of vascular disease [44,50]. The CRP in such cases is probably a marker for the presence of the pro-inflammatory cytokines which are more directly involved in the pathogenesis of vascular disease through their promotion of insulin resistance, dyslipidemia and inflammatory macrophage activity. IL-1, IL-6 and TNF are consistently associated with these mediators of vascular pathology [51]. More recently the pro-inflammatory cytokines interleukin-15 (IL-15), interleukin-18 (IL-18) and interleukin-8 (IL-8) have been found to be more specific risk factors for coronary artery disease, diabetes and some cancers [52,53,54]. IL-1 and TNF also have a suppressive effect on some hepatocyte synthetic functions [55,56]. The main manifestation of this in clinical practice is a reduction of the serum albumin concentration, which is a useful inverse indicator of systemic inflammation.

Receptors for pro-inflammatory cytokines are found on the surface of various types of neurons [57]. The evidence is building for a role of pro-inflammatory cytokines, and other substances involved in inflammation such as cortisol and adrenalin, in the modulation of neuronal function. Receptors for several cytokines are also present on brain neurons and glial cells, including TNF, IL-1, IL-6 and IL-1ra, and blood-brain barrier transport systems have been demonstrated that enable a number of cytokines to enter the cerebro-spinal fluid and brain interstitial fluid, including IL-1, TNF, IL-6 and IL-1ra, either directly or indirectly. In animal models, it has been shown that measurable changes of behavior, such as anorexia, hiding and sleepiness, are induced by exposure to IL-1 and TNF [57,58,59]. These phenomena provide a route to an understanding of the chemical-anatomical mechanisms whereby inflammation affects central nervous system function, including a substrate for lethargy and delirium. Most research attention has been given to dementia, particularly AD, and there is no doubt that the inflammatory milieu, including cytokines, is altered in AD [30,60]. What is less clear is the role of cytokines in the initiation of the process leading to AD, or in its progression; are the measured cytokine changes merely a reactive consequence or a key pathogenic component? The apparent protective effect of regular exercise on cognitive function suggests the latter [61]. There is evidence that the autonomic dysfunction observed during and after acute inflammatory illnesses such as pneumonia is, at least in part, mediated by pro-inflammatory cytokines [57]. The impairment of cardiovascular autonomic reflexes, an important contributory cause of the orthostatic hypotension often found in older patients after acute illnesses, is probably to a significant extent a result of cytokine-mediated down-regulation and is therefore of clinical importance [62]. The same or a similar mechanism is likely to be the reason for the persistent cardiovascular autonomic dysfunction observed in chronic inflammatory conditions. The role of inflammatory chemistry in sensory, motor and co-ordination dysfunction is much less clear and therefore an attractive target for future research.

The adverse influence of pro-inflammatory conditions on the structure and function of skeletal muscle appears closely bound with IL-6, which is dealt with in the next paragraph.

## 7. Skeletal Muscle, Exercise, Inflammation and the Role of IL-6

IL-6 has been mainly considered to be a pro-inflammatory cytokine with a broad range of effects including augmentation of IL-1 and TNF, and as an inducer of obesity-related insulin resistance in inactive subjects. Indeed, blood IL-6 levels do rise early in response to acute pro-inflammatory stimuli and are usually elevated above baseline in chronic inflammatory states. However, the role of IL-6 in the innate immune network is clearly much more complex. The main source of IL-6 is contracting skeletal muscle and it is therefore often sub-classified as a myokine [63], though it is also produced by a range of other cells. In the context of exercise, it has been shown to have autocrine, paracrine and endocrine functions that mediate insulin-sensitizing effects, and in that metabolic context it promotes the release of anti-inflammatory cytokines such as IL-10 [64]. The full metabolic action of IL-6 is unknown and it seems to have an effect on inflammatory chemistry that is conditional upon prevailing conditions in a pleiotropic manner [63,64]. Therefore, during sepsis IL-1 promotes IL-6 and TNF whereas after exercise IL-6 promotes IL-10 and interleukin-1 receptor antagonist (IL-1ra). This apparent orchestrating function of a cytokine produced and released by active skeletal muscle puts it in a central position when considering the effects of exercise on inflammation, and it also raises the question as to whether IL-6 should be regarded not only as an inflammatory cytokine but also as an autocrine and paracrine hormone [65]. Other myokines appear to have more specific functions than IL-6 and less overall influence on the setting of the immune web. The apparent pleiotropic behavior of IL-6 also indicates the possibility that another, as yet unidentified, substance is a more proximal controller. However, for this review IL-6 will be positioned as a pleiotropic cytokine with an apparently key modulating role in immune responses, particularly in the context of exercise.

The rise in IL-6 during and immediately after exercise occurs at all ages [44,60,61,66,67]. Though most clearly demonstrated during resistive rhythmic concentric exercise, such as cycling, IL-6 is also released during eccentric and isometric exercise [68]. Of course, normal day to day physical activity tends to be a variable mix of all three patterns of muscle use. The clearest studies have been performed on young adults undergoing vigorous exercise. Rises of IL-6 in peripheral blood of 100-fold or greater above baseline have been shown in young adults after prolonged exercise at high work rates, such as competitive long-distance running [69]. A dose-response relationship has been observed with work rate and duration, particularly the latter. Measurable rises have been shown after moderate exercise, such as cycling or brisk walking for 10–20 min, including in old age [70]. However, there are no studies of the IL-6 response to the very mild exercise, such as slow walking for a few minutes, that often represents the maximum achievable work rate of frail elderly people. The IL-6 response to exercise is further complicated by an apparent reduction in baseline levels between episodes of recurrent exercise [71]. In those who exercise regularly it can be argued that the fall in baseline IL-6 between bouts of exercise might be the key anti-inflammatory consequence of muscle activity, rather than the insulin-sensitizing effect of transient peak post-exercise levels [72]. The autocrine and endocrine effects of IL-6 on myocytes also appear to depend on the pattern and context of IL-6 release so that the acute peaks that occur with vigorous exercise appear to have a role in building muscle bulk, contractile strength and endurance whereas chronically elevated baseline IL-6 levels are thought to play a part in the degenerative changes that lead to myocyte apoptosis and sarcopenia [66,73]. A reasonable interpretation might therefore be that the IL-6 mediated benefits of exercise are due to at least 2 mechanisms. First, a general anti-inflammatory effect through the stimulation of IL-10, interleukin-4 (IL-4) and IL-1ra that could have a positive but probably indirect influence on, for example, endothelial function, insulin sensitivity and hepatic synthetic function. Second, mechanisms whereby high peak exercise-induced levels improve muscle structure and performance and lower baseline levels reduce the tendency to sarcopenia, most likely through poorly understood gene-switching mechanisms that control myocyte metabolism and apoptosis [11,74,75,76].

## 8. Exercise to Help Prevent, Delay or Ameliorate Frailty

A number of large high quality studies have established the beneficial effects of regular moderately resistive aerobic exercise, such as walking and cycling. This has been established from an epidemiological perspective, based on self-reported exercise habits, and during supervised intervention studies. The methods used for data collection and analysis vary across the published literature but the overall findings show an independent effect of exercise in reducing the risk of all-cause mortality, stroke, cardiovascular disease and type 2 diabetes [77,78]. There is also evidence of a reduced risk of sarcopenia [79], dementia, depression and physical dependency [20,58,80]. Many of these conditions fall within the definition of frailty or are contributing factors to frailty and sarcopenia. People who take regular exercise also report higher levels of wellbeing in intervention studies [81]. Further, more specific exercise-based interventions are of proven value in COPD [82] and after myocardial infarction [83]. Though some benefits are dependent on the intensity of an exercise program, for example building cardiovascular endurance, it has recently been shown that brief, low-intensity, exercise such as 20 min walking per day has a positive effect on all-cause mortality, particularly in individuals with obesity [77], a known pro-inflammatory state. Relatively few exercise studies have focused specifically on elderly people but the evidence as it stands indicates positive effects on mobility, function and dependency, and probably a reduction in stroke and all-cause mortality [20,39]. Of course, some of the described benefits of exercise are likely to be mediated by non-cytokine mechanisms, including psychological effects, neuro-humoral changes and through the release of other hormone-like substances such as endorphins.

Having established a beneficial effect of exercise on a number of health indicators and outcomes, and given the association and probable mechanistic relationship between those indicators and a chronic pro-inflammatory state, the review will now consider the effect of exercise on the chemistry of inflammation.

## 9. Exercise to Reduce the Blood Markers of Inflammation in Old Age

Several observational studies employing good methodology have looked at the effect of exercise on various biochemical blood markers of inflammation in older adults. Most of the data pertain to the 60–79 age range. A consistent inverse dose-related correlation between physical activity and inflammatory biomarkers was found, with some variation related to gender, use of supplements, nutritional state and body composition [20,84,85]. For example, inactive older people were found to have around 2-fold higher mean blood CRP and IL-6 levels compared to matched moderately active people. In the same study those with mean baseline CRP and IL-6 levels in the lower quartile were shown to have walking speed and grip strength in the upper quartile, though there was a wide range of individual variation in the blood markers of inflammation [85].

The Cardiovascular Health Study [86] and the British Regional Heart Study [87] included a total of more than 10,000 people over the age of 60. These studies showed an inverse relationship between CRP and self-reported physical activity that persisted after adjustment for multiple risk factors. The relationship held even at estimated exercise-related energy consumption levels as low as 368–1050 kcal/week. Most studies also showed a fall in TNF, IL-1 and baseline IL-6, when measured [84,85] though the effects on cytokines were more consistently found in studies using relatively strenuous exercise [85,88]. One large study comparing sedentary older people with those undertaking regular light and moderate exercise found mean CRP levels to be 0.43 and 0.73 mg/L (*p* = 0.024 and <0.001) respectively lower in the active groups and significantly lower levels of IL-6 and TNF in the moderate group (−0.33 pg/mL, *p* = 0.14 and −0.31 pg/mL, *p* = 0.03 respectively) [88]. Light exercise, such as slow walking and intermittent housework, were only clearly associated with a fall in CRP [89]. At lower exercise levels, the fall in CRP was more consistent in elderly men, even after adjustment for body mass index (BMI), though the magnitude of the change was small and similar to that seen after treatment with statins [20,88]. Adiposity has been shown to correlate positively with baseline CRP, and some studies have found no response to mild exercise once changes in body composition are taken into account [20,90,91]. Of course, adipose tissue has a pro-inflammatory effect, so the apparent anti-inflammatory effect of exercise might be indirect in those circumstances.

There are few data concerning exercise performance or measures of aerobic fitness and inflammatory biomarkers in older adults, and none that used objective laboratory measures of cardio-respiratory capacity such as anaerobic threshold or maximum oxygen uptake. The findings in old age have been equivocal, and difficult to interpret due to different methodologies and definitions. One study found an inverse relationship between maximum walking speed and baseline IL-6 and CRP [85], while another found that the relationship disappeared when adjustment was made for estimated body fat [92].

## 10. Exercise to Avoid or Delay Frailty

The available data suggest that older people who are able to perform regular moderate exercise as part of a lifestyle habit can reduce their risk of a number of adverse health outcomes, including some of the components of frailty and sarcopenia. This benefit is probably due to the anti-inflammatory and hormone-like effects of IL-6 release from skeletal muscle and other cytokine mediated mechanisms. The benefits have been shown to include improved endothelial-cardiovascular function, reduced risk of certain cancers, conservation of skeletal muscle strength, independent function, cognition and mood [93]. The optimal dose of exercise in older age is not known. The potential benefits of moderate exercise have been described above, and while there is no consistent evidence that exercise regimens with more demanding work rates or prolonged duration should be discouraged, older individuals should be made aware of the risks as well as the benefits.

## 11. When Exercise is Not Appropriate

There are clearly circumstances when exercise cannot be realistically used to suppress systemic inflammation or offset frailty, for example, in some post-traumatic or post-surgical orthopedic conditions when weight-bearing exercise is contraindicated. Also, for many very frail older patients, and for those with disabilities that make exercise regimens impractical, it is not possible to reach the work rates needed to achieve and maintain an anti-inflammatory benefit. This limitation is also true for many elderly patients during the early recovery phase of acute pro-inflammatory states such as sepsis and trauma. Geriatricians are very familiar with the frail post-septic patient who is unable or reluctant to engage in attempts to regain basic mobility let alone undertake a graduated exercise program. Clinicians need to consider alternative means to return patients to a baseline state of innate immunity in an attempt to lessen the impact of sustained inflammation. Assuming that nutritional factors have been optimized, the original stimulus for inflammation, such as infection, has been treated and a reasonable attempt has been made to encourage physical activity, the next interventions to consider in future are likely to be pharmacological. Of course, it must be emphasized that drugs should not be seen as a replacement for other measures, and a case can be made for adding immune-modulating drugs to other treatments, such as exercise, when that is possible. Many drugs are known to modulate the innate immune network in an anti-inflammatory direction, though clinical studies are sparse. The remainder of this paper will consider the evidence for whether drug treatments for inflammation might reduce the progression to frailty, and in the acute setting possibly prevent it.

As has been described in detail above there is a substantial body of evidence that indicates the involvement of systemic inflammation in the pathogenesis of several aspects of the frailty syndrome in old age. This is a complex field that spans clinical medicine, immunology, pathology and biological chemistry, and there is a fragmentary understanding of the regulatory mechanisms involved, the range of normative states and the variations over time. However, for the purposes of this paper we are concerned with the possibility of using drugs to modify two of the disordered immune patterns that are encountered in old age:

First, chronic low-amplitude inflammation, as described earlier in the paper. Second, acute inflammation with inappropriate resolution. This is a less well recognized but not uncommon response to ostensibly ordinary infections, such as pneumonia, urinary tract infections, Gram-negative septicemia and cellulitis, or other physiological insults such as accidental or surgical trauma. As described above, in many elderly people the biochemical and clinical pro-inflammatory response to acute infection does not resolve as quickly as it does in younger adults. The rises in IL-1, TNF, IL-6 and CRP persist longer, and the rise in anti-inflammatory IL-10 is delayed and also prolonged [94]. In such older patients, the pro-inflammatory clinical phenotype is consequently often prolonged [22,46,95] and is associated with a number of clinically important consequences such as delirium, lethargy, low mood, autonomic dysfunction, reduced appetite, weakness and increased dependency. There is a slow, and sometimes incomplete, return to baseline. Geriatricians and other clinicians involved with the care of elderly people are becoming increasingly familiar with this phenomenon and the adverse effect it can have on a range of outcomes for their patients.

Healthy elderly volunteers exposed to endotoxin showed an approximate 2-fold longer time for biochemical markers of inflammation, such as TNF and IL-1, to return to baseline [21,46]. The evidence also indicates that frailer elderly patients, with higher levels of background inflammation, have an even more extended period of persisting inflammation after acute infections [46]. The clinical correlates for that phenomenon include persisting anorexia, low mood and lethargy, incomplete resolution of delirium and CRP levels that do not return fully to the normal range. This suggests an impairment of regulatory anti-inflammatory function in old age that is a likely substrate for slower clinical recovery resulting from an extended exposure to the clinically negative effects of certain pro-inflammatory cytokines, particularly TNF and IL-1, and is an attractive target for drug treatments.

## 12. The Rationale for Pharmacological Interventions

As mentioned above, it is evident that in clinical practice there will be a large number of people who are not able to take part in exercise programs to reduce inflammation, ameliorate sarcopenia or delay frailty in either the acute or chronic contexts. This paper will now discuss the potential for a range of drugs to be utilized to modulate patients’ immune status from a deleterious inflammatory phenotype toward a less inflamed surveillance phenotype. In most cases the precise mechanisms are not fully resolved, and because of the complex web-like interactivity of immune cells, cytokines and other immune system bio-chemicals it is probably not appropriate to attempt to visualize the system in terms of conventional linear reaction models. Certain drugs appear to pull the immune web in an anti-inflammatory direction; a phenomenon that has considerable therapeutic potential.

The evidence for efficacy is in many instances based on clinical observation, animal and in vitro experiments and small-scale clinical studies. Adequately powered prospective clinical trials with good methodology are almost non-existent in the field of drug interventions for frailty and sarcopenia. However, in some cases there is strong enough evidence to justify the use of drugs as an “unlicensed indication”. This paper will now look at a selection of drugs that might be used for this purpose, concentrating on the methyl-xanthine drug theophylline which probably has the best supporting evidence and is an attractive candidate for further clinical research.

## 13. The Anti-Inflammatory Effects of Methyl-Xanthines

Theophylline, a methyl-xanthine phosphodiesterase inhibitor drug, has been used as a treatment for asthma and COPD for many years. Its usefulness as a bronchodilator is limited by the need to maintain plasma concentrations in a therapeutic range (10–15 mg/L) that is close to the toxic range (>20 mg/L) and its clinical utility has been overshadowed by the superior performance of beta-2 agonist and anti-muscarinic bronchodilators. However, it was observed that certain favorable patient outcomes, such as walking performance, were reported in patients with COPD given theophylline treatment even when little or no measurable change occurred in spirometry indices of airways obstruction or arterial blood gas tensions, and the effect was also observed at low (<10 mg/L) plasma levels [96]. Further research demonstrated anti-inflammatory properties of theophylline locally on airways inflammation, systemically and in vitro [96,97]. Other methyl-xanthine drugs such as pentoxifylline have similar properties [98] but the body of evidence is greatest for theophylline. The complete molecular mechanism of the anti-inflammatory properties of theophylline remains unknown, and probably varies depending on the pathophysiological context. One study showed that theophylline at an in vitro concentration of 15 mg/L reduced mean TNF secretion from 0.26 pg/mL to 0.21 pg/mL (*p* < 0.05) by peripheral blood monocytes and increased mean IL-10 release from 0.35 pg/mL to 0.98 pg/mL (*p* < 0.01). It also reduced mean interferon-gamma concentrations from 24.5 pg/mL to 13.4 pg/mL (*p* < 0.05) [99]. Similarly, in vivo exposure to comparable concentrations of pentoxifylline caused a progressive reduction in the production of pro-inflammatory IL-1, IL-6, IL-8 and TNF of between 20 and 80 percent by harvested peripheral blood monocytes over 4 days [98]. This is probably mediated through an epigenetic mechanism that includes induction of histone deacetylase-dependent gene switching toward a more anti-inflammatory phenotype in immune cells rather than by phosphodiesterase inhibition [97,100]. Theophylline also reduces baseline IL-6 levels in peripheral blood [101]. IL-6 however, has a complex role that is dependent on physiological context, such that the sharp transient rise in IL-6 that occurs after exercise appears to have an anti-inflammatory effect, possibly mediated by IL-10 release but also directly through an up-rating of insulin receptor sensitivity and an influence on skeletal myocytes that slows or delays atrophy [63,64,102,103]. On the other hand, the higher resting trough IL-6 levels that are associated with chronic and acute pro-inflammatory states and trauma appear to increase the risk of delirium [104] and probably predispose to sarcopenia [71,72,73,105,106,107]. There is no evidence regarding the effect of theophylline or similar drugs on post-exercise IL-6. The mechanism of these effects appears to be due to theophylline-induced re-direction toward the anti-inflammatory state via gene switching in macrophages and other immune cells which have been shown to have several dose-dependent gene switches that up- and down-regulate various cytokines [100,108,109]. An interesting observation is that subjects with COPD treated with the addition of theophylline to a standard regimen had lower baseline CRP levels and better functional scores compared with control subjects [110]. These effects have been described as immune “modulation” by some authors as it appears that theophylline at currently used therapeutic doses tends to reduce inflammation without compromising the protective effect of an appropriate acute inflammatory response to infection, and a recent study has shown a reduction in mortality in patients with severe sepsis treated with theophylline in a critical care setting [111,112]. Importantly, the potentially beneficial anti-inflammatory effect also occurs at in vitro theophylline concentrations well below the level needed for effective broncho-dilatation. In one study IL-6 and IL-8 production by fibroblasts was reduced by 25–30 percent at a theophylline concentration of 5 mg/L [113]. At these lower concentrations (5–10 mg/L) it is unusual to encounter side effects or signs of theophylline toxicity, such as tachycardia, cardiac arrhythmias, tremor, nausea, diuresis and sleep disturbance.

Therefore, a case can be made for the use of low-dose theophylline to modulate inflammation when it is inappropriately prolonged after stimuli such as sepsis and trauma in frail elderly patients, particularly when they have clinical and laboratory features of extended inflammation and are unable to become physically active. There might also be a role for the long-term use of theophylline to dampen chronic inflammation to reduce the progression to overt frailty. There is a clear need for properly conducted clinical trials to measure the effects of theophylline on defined outcomes. The main targets for further study should be placebo controlled trials of low-concentration (5–10 mg/L) theophylline, given orally, as adjunctive treatment in elderly patients recovering from sepsis or trauma, and to establish whether chronic low-grade inflammation can be modified. Studies in mobile and immobile patients should be considered. In all study designs defined outcomes would need to include mortality, mobility scores, functional scores, measurements of muscle strength, cognition and wellbeing scores. Utility measures, such as length of hospital stay, should also be included. Of course, a full consideration of potential research questions is outside the scope of this paper. As the evidence currently stands, clinicians should consider treating suitable patients with low-dose theophylline as an “unlicensed indication” until trials have been conducted.

## 14. Other Drugs with Immune-Modulating Properties

Though methyl-xanthines are arguably the most promising immune modulating drugs in the context of frailty, drugs in several other classes also influence the innate inflammatory response, though in many instances the documented side effects reduce their potential for use in the context of old age.

Monoclonal antibodies (MCAs) have been used with great success in inflammatory diseases, with anti-TNF drugs, such as infliximab, for rheumatoid arthritis being one of the more ubiquitous examples. However, MCAs have the disadvantage of being highly specific for their target cytokines or cytokine receptors [114] and therefore appear to have a powerful but narrower influence on immune settings. They also tend to over-suppress some aspects of immune surveillance and predispose to opportunistic infection and some malignancies, particularly lymphomas, though the latter seems only to occur after prolonged treatment. They are also expensive, have to be given by injection and require close monitoring, all of which add further limitations.

Corticosteroids have a long track record of use as broad-spectrum anti-inflammatory drugs that act by down-regulation of pro-inflammatory immune cell behavior rather than by re-establishing the baseline equilibrium of the innate immune system [115]. When used systemically, they are limited by their mineralocorticoid effects (sodium and water retention), glucocorticoid effects (enhanced protein catabolism and glucose intolerance), tendency to cause increased wakefulness and precipitate delirium, and suppression of cell-mediated immunity.

Thalidomide and related drugs are established as anti-inflammatory agents with clinical uses in the treatment of leprosy, other mycobacterial diseases, and as an adjunctive drug in certain oncology chemotherapy regimens. Thalidomide and related drugs appear to have subtle immune modulating properties that include suppression of pro-inflammatory cell behavior, and a reduction of IL-1, IL-6 and TNF release [116,117]. This multi-positional tug of the immune chemical web away from an inflammatory state is similar to that of methyl-xanthines. Thalidomide itself is limited by a wide range of side-effects at therapeutic doses, but a recent study of a related drug that has fewer side effects, diamino-diphenyl sulfone, has found less evidence of sarcopenia in elderly patients treated with it for leprosy [118]. A prospective trial would now be justified and informative.

4-aminoquinoline drugs (chloroquine, hydroxychloroquine and amodiaquine) have a suppressive action on the release if IL-1, IL-6, TNF and interferon gamma and tend to promote an increase in the population of immune cells producing IL-10 and IL-4 [119]. They have been used as anti-inflammatory drugs in certain auto-immune disorders such rheumatoid arthritis and systemic lupus erythematosus. The effects of low doses on cytokine dynamics are not known and at usual therapeutic doses a high proportion of patients report unpleasant side effects, though anti-inflammatory doses are usually lower than those used for the treatment of malaria. This class of drugs deserves further scrutiny in the management of sarcopenia and frailty.

Non-steroidal anti-inflammatory drugs reduce systemic inflammation and there is evidence from at least one high quality study that they have a useful effect in older patients to preserve muscle function [120]. They are limited, but not excluded, from long term use by their nephrotoxic, gastropathic and pro-coagulant side effects. Perhaps further research needs to be planned to determine whether the beneficial effect on muscle is preserved at doses lower than those used for anti-inflammatory analgesia, in which case the adverse effects would be expected to be a lesser problem.

Beta-adrenergic receptor blockers, such as propranolol and metoprolol, have been found to alter innate immune responses, with a tendency toward an anti-inflammatory shift. This has largely been observed in relation to the inflammatory profile seen in chronic heart failure and atheromatous vascular disease [121,122]. The mechanism is probably by adrenergic receptor blockade and a consequent reduction in catecholamine-mediated release of pro-inflammatory cytokines by monocytes. Some of the reduction in all-cause mortality observed in patients treated with beta-blockers for cardiac conditions might be due to systemic anti-inflammatory effects.

Statins have an anti-inflammatory effect that appears to be independent of, or not directly related to, the induced changes in plasma lipids. The reduction in endothelial inflammation, probably mediated by a protective effect of statins on TNF-induced increases in reactive oxygen species in endothelial cells, has received most attention, but there is evidence of a more systemic effect [123]. However, there have been no studies of the effect of statins on acute-phase inflammation in the context of sepsis and trauma.

The biguanide drug metformin, has been shown to have anti-inflammatory properties, probably mediated through switching macrophages to a more anti-inflammatory phenotype. Most of the evidence is centered on in vitro studies of endothelial function. The improved vascular outcomes observed in patients taking long-term metformin are likely to be partly due to anti-inflammatory action [124,125]. However, there are no studies of its effect in acute inflammation or age-related chronic inflammatory states other than in the context of diabetes.

Hormonal treatments employing growth hormone analogues, female hormone replacement therapy, testosterone and adrenocorticotrophic-releasing factor analogues have been explored as potential treatments for sarcopenia [126], though the evidence for efficacy in very old and frail patients is sparse. These treatments are more likely to have direct actions on myocytes rather than an indirect action through alterations of the inflammatory network.

## 15. Conclusions

There is a rapidly growing understanding of the part played by systemic inflammation in the promotion of frailty in older people, including sarcopenia. Regular moderate resistive exercise has been shown to improve a number of health indicators, ameliorate the progression to sarcopenia, reduce the expression of the frailty phenotype and support independent function. One of the main mechanisms for this effect is probably a direct result of the property of exercise to shift the immune network to a less pro-inflammatory setting. Through this mechanism, and possibly others, exercise is therefore an important factor in preserving health and in the recovery from illness and trauma. However, some patients are not able to achieve the work rates or maintain the long-term exercise programs that lead to those benefits. In such patients, there is theoretical, laboratory, observational and some clinical evidence that a number of drugs with immune modulating properties could be used to reduce the pro-inflammatory state, with potential benefit to wellbeing, physiological performance and independent function. Such drugs should also be considered as adjunctive treatments for patients who can perform exercise. There is a need to subject such drugs, such as theophylline, to properly conducted trials to explore that therapeutic possibility.

Therefore, the main messages for geriatricians and other clinicians are:

- Chronic inflammation and prolonged post-acute inflammation predispose to frailty and sarcopenia.
- Whenever possible, the causes of inflammation, such as infection, should be treated promptly and nutrition should be optimized.
- Moderate exercise reduces inflammation and improves a wide range of health outcomes, and should be encouraged in as a preventive strategy and as part of the treatment for pro-inflammatory conditions.
- Drugs with immune modulating properties, such as theophylline, should be considered as adjunctive treatment for systemic inflammation, including those able to exercise, and might be particularly helpful for patients who are unable to take part in an exercise program.
- Clinical trials are needed to establish the role of anti-inflammatory drugs in this clinical context.
